# Chk1 Inhibition Restores Inotuzumab Ozogamicin Citotoxicity in CD22-Positive Cells Expressing Mutant p53

**DOI:** 10.3389/fonc.2019.00057

**Published:** 2019-02-18

**Authors:** Elena Tirrò, Michele Massimino, Chiara Romano, Maria Stella Pennisi, Stefania Stella, Silvia Rita Vitale, Annamaria Fidilio, Livia Manzella, Nunziatina Laura Parrinello, Fabio Stagno, Giuseppe Alberto Palumbo, Piera La Cava, Alessandra Romano, Francesco Di Raimondo, Paolo G. Vigneri

**Affiliations:** ^1^Department of Clinical and Experimental Medicine, University of Catania, Catania, Italy; ^2^Center of Experimental Oncology and Hematology, Azienda Ospedaliero-Universitaria “Policlinico-Vittorio Emanuele”, Catania, Italy; ^3^Department of Drug Sciences, University of Catania, Catania, Italy; ^4^Division of Hematology, Azienda Ospedaliero-Universitaria “Policlinico-Vittorio Emanuele”, Catania, Italy; ^5^Department of Medical, Surgical Sciences and Advanced Technologies “G. F. Ingrassia”, University of Catania, Catania, Italy; ^6^Department of Surgery and Medical Specialties, University of Catania, Catania, Italy

**Keywords:** p53, inotuzumab ozogamicin, Chk1, B-ALL, antibody-drug conjugates

## Abstract

Inotuzumab ozogamicin (IO) is an anti-CD22 calicheamicin immunoconjugate that has been recently approved for the treatment of relapsed or refractory B-Acute Lymphoblastic Leukemia (r/r B-ALL). We employed both immortalized and primary cells derived from CD22-positive lymphoproliferative disorders to investigate the signaling pathways contributing to IO sensitivity or resistance. We found that the drug reduced the proliferation rate of CD22-positive cell lines expressing wild-type p53, but was remarkably less effective on cells exhibiting mutant p53. In addition, CD22-positive cells surviving IO were mostly blocked in the G2/M phase of the cell cycle because of Chk1 activation that, in the presence of a wild-type p53 background, led to p21 induction. When we combined IO with the Chk1 inhibitor UCN-01, we successfully abrogated IO-induced G2/M arrest regardless of the underlying p53 status, indicating that the DNA damage response triggered by IO is also modulated by p53-independent mechanisms. To establish a predictive value for p53 in determining IO responsiveness, we expressed mutant p53 in cell lines displaying the wild-type gene and observed an increase in IO IC_50_ values. Likewise, overexpression of an inducible wild-type p53 in cells natively presenting a mutant protein decreased their IC_50_ for IO. These results were also confirmed in primary CD22-positive cells derived from B-ALL patients at diagnosis and from patients with r/r B-ALL. Furthermore, co-treatment with IO and UCN-01 significantly increased cell death in primary cells expressing mutant p53. In summary, our findings suggest that p53 status may represent a biomarker predictive of IO efficacy in patients diagnosed with CD22-positive malignancies.

## Introduction

In the adult population, Acute Lymphoblastic Leukemia (ALL) is an uncommon hematological disorder (0.4% of all new cancer cases in the US) characterized by highly proliferative immature lymphoid progenitors usually derived from the B-cell lineage (1, 2).

While B-ALL treatment using the association of vincristine, dexamethasone, cyclophosphamide or anthracyclines (3) generates a complete remission in 80–90% of patients (4), 5-year overall survival (OS) rates remain in the 40% range, plummeting to <10% in case of disease relapse (5). To improve this dismal outcome, several targeted therapies have been recently developed including tyrosine kinase inhibitors (TKIs)—for Philadelphia-positive (Ph+) variants of the disease (6)—and monoclonal antibodies targeting B-cell membrane receptors such as CD19, CD20, CD22, and CD52 (5, 7).

Human CD22 is a surface antigen expressed in pro-B and pre-B cells that heavily contributes to the regulation of B-cell function (8). After ligand binding, CD22 undergoes constitutive internalization followed by lysosomal degradation (9). Hence, this surface antigen represents an ideal target to kill leukemic B-cells using antibody-drug conjugates (ADCs) that combine the antibody specificity for a selected antigen with the cytotoxicity ability of different cell-killing agents (10).

Inotuzumab ozogamicin (IO), also known as CMC-544, is a highly specific ADC targeting CD22-positive lymphoproliferative diseases. IO consists of a semi-synthetic derivative of N-acetil-γ calicheamicin 1,2-dimethyl hydrazine dichloride (CalichDMH) covalently linked—via an acid-labile 4-(4'-acetylphenoxy) butanoic acid—to a humanized monoclonal IgG4 anti-CD22 antibody (11, 12). CalichDMH derives from the actinomyces Micromonospora echinospora, subspecies calichensis, and its cytotoxicity relies on the ability to bind the minor groove of the DNA helix producing double strand breaks (13, 14). In turn this DNA damage arrests the cell cycle in G2/M, activating multiple apoptotic mechanisms (15, 16). The Chk1/2 and the p53 signaling pathways have both been implicated in maintenance of the G2/M arrest triggered by DNA damage as the former proteins, upon induction by ATM (Ataxia Telangiectasia Mutated Kinase) and/or ATR (Ataxia Telangiectasia And Rad3-Related Protein) up-regulate 14-3-3 proteins or lead to p21 transactivation. In both circumstances, the net biological result of these events is prolongation of the G2/M arrest via 14-3-3-dependent cytoplasmic sequestering of Cdc25C or p21-dependent regulation of Retinoblastoma protein (17).

The p53 protein—encoded by *TP53* gene - plays a pivotal role in modulating DNA damage response, cell proliferation, differentiation, and death (18, 19). Most p53 mutations result in protein loss of function and, if coupled with deleterious alterations involving the p53 region of the remaining allele, favor cellular oncogenic transformation. These non-synonymous p53 mutations usually occur in the DNA binding domain encoded by exons 5–8 of the *TP53* gene. As a result, p53 protein structure is disrupted and p53 can no longer bind to its target genes and exert its transcriptional activity (20, 21).

In adult B-ALL, the most commonly reported *TP53* alterations are missense mutations that, while infrequent, are usually associated with a poor outcome (22). Furthermore, the incidence of *TP53* mutations increases at disease relapse and has been frequently reported in adult ALL that does not display recurrent fusion genes (23).

IO has been recently approved for the treatment of adult patients with relapsed or refractory CD22-positive B-ALL (24) or adult patients with Ph+ ALL that have failed treatment with at least one TKI (25, 26), showing significantly higher remission rates than standard therapy.

In the present study we investigated the role of p53 in modulating the IO responsiveness of both immortalized and primary CD22-positive B-ALL cells.

## Materials and Methods

### Immortalized Cells

Burkitt lymphoma (BL-2, Namalwa, Raji, and Ramos), ALL (SUP-B15) and Acute Myeloid Leukemia (HL-60) cell lines were obtained from the German Collection of Microorganisms and Cell Cultures DSMZ and used for fewer than 6 months after receipt.

BL-2, Namalwa, Raji, Ramos, and HL-60 cells were maintained in RPMI-1640 medium while SUP-B15 were grown in Mc-Coy 5A medium (both from Sigma-Aldrich). Media were supplemented with 10% (Namalwa, Raji and HL-60) or 20% (BL-2, SUP-B15 and Ramos) heat-inactivated fetal bovine serum (FBS) (Euroclone), 2 mmol/L L-glutamine (Sigma-Aldrich) and penicillin/streptomycin (100 U/mL and 50 μg/mL, respectively, also from Sigma-Aldrich).

Human bone marrow-derived mesenchymal stem cells (MSCs) immortalized by forcing the expression of telomerase reverse transcriptase (TERT) (donated by Dario Campana, Department of Pediatrics, Yong Loo Lin School of Medicine, National University of Singapore) were grown in RPMI-1640 medium supplemented with 10% FBS, 2 mmol/L glutamine, 10^−6^ M hydrocortisone (Sigma-Aldrich), 100 U/mL penicillin and 50 μg/mL streptomycin as previously described (27). Immortalized MSCs were seeded in 96-well plates coated with 1% gelatin (Sigma-Aldrich) and grown until they reached confluence. Before seeding primary cells, the RPMI-1640 medium was removed from MSCs and cells were washed seven times with AIM-V medium (Thermo Fisher Scientific) to remove FBS and hydrocortisone. All cell lines were maintained in an incubator set at 37°C with 5% CO_2_.

### Primary Cells

Bone marrow (BM) samples were collected from six patients with newly diagnosed B-ALL and four refractory—relapsed B-ALL (r/r B-ALL) according to the 2008 WHO criteria. Patients were followed in the Division of Hematology of the A.O.U. Policlinico—Vittorio Emanuele and signed an informed consent releasing anonymously their samples for research purposes in accordance with the Declaration of Helsinki. Only subjects with neoplastic cells expressing >80% CD22-positive were eligible for this study.

BM mononuclear cells were isolated by Ficoll-Paque Premium (GE Healthcare) density-gradient centrifugation according to the manufacturer's protocol.

For apoptosis evaluation, BM mononuclear cells were maintained in AIM-V medium and seeded onto immortalized MSC cells. Primary cells were instead plated in AIM-V medium in stroma free wells to calculate the 50% inhibitory growth concentration (IC_50_) for the drugs employed in the study.

### Immunophenotype Analysis of Immortalized Cell Lines

The expression of surface markers of immortalized cell lines was determined by flow cytometry using the following monoclonal antibodies: anti-CD22 Fluorescein isothiocyanate (FITC) (Clone SJ10.1H11) and anti-CD33 Phycoerythrin (PE) (clone D3HL60.251) (both from Beckman Coulter). Cell lines were stained according to the manufacturer's instructions and analyzed by flow-cytometry using Cytomics FC500 (Beckman Coulter). For each condition, 10.000 events were acquired. Results were expressed as the percentage of CD22- or CD33-positive cells over total number of analyzed events.

### Chk1 and p53 Constructs and Mutagenesis

To achieve the inducible pTRIPZ short-hairpin RNA (shRNA) anti-Chk1 constructs we moved human shRNAs anti-Chk1 (cat. n° RHS4531, Dharmacon/Horizon Discovery Ltd) from the pGIPZ to the pTRIPZ vector according to the manufacturer's protocol.

To obtain different p53 constructs we employed the following strategies. The human p53-EGFP sequence was first excised with NheI and NotI restriction enzymes from the pEGFP-N1-p53 plasmid (a gift of Prof. Francesco Frasca, Division of Endocrinology, Department of Clinical and Experimental Medicine of the University of Catania) and then cloned in the pcDNA3.1 expression vector (Thermo Fisher Scientific).

To obtain the p53^R248Q^ mutant, the pcDNA3.1-p53-EGFP plasmid was subjected to a mutagenesis reaction using the Quick-Change II XL Site-Direct Mutagenesis Kit (Agilent Technologies) as elsewhere specified (28). Primers employed to generate the required mutations in the p53 wild-type sequence were 5′-GGCGGCATGAACC**AGA**GGCCCATCCTC-3′ (forward) and 5′-GAGGATGGGCC**TCT**GGTTCATGCCGCC-3′ (reverse). Codons indicated in bold mutagenize Arginine (R) in position 248 in Glutamine (Q). Proper incorporation of the desired mutation was verified by Sanger sequencing. Afterwards, the p53^R248Q^-EGFP sequence was excised from pcDNA3.1 with XhoI and cloned in the pLEX lentiviral vector (Open Biosystem).

To create a human inducible p53 wild-type-EGFP construct, p53 and EGFP were separately cloned in the inducible pTRIPz vector (Open Biosystem). Initially, p53 was amplified from the pcDNA3.1-p53-EGFP vector using the indicated forward 5′-CGCACCGGTGCCACCATGGAGGAGCCGCAGTCAGA-3′ and reverse 5′-CCGCTCGAGGTCTGAGTCAGGCCCTTCTG-3′ primers and cloned AgeI-XhoI in pTRIPz generating the pTRIPz-p53 plasmid. Subsequently, EGFP was amplified from the pEGFP-N1 plasmid employing the indicated forward 5′-CCGCTCGAGATGGTGAGCAAGGGCGAGGA-3′ and reverse 5′-CGACGCGTCCTAGGTAATACGACTCACTATAGGGTTACTTGTACAGCTCGTCCATG-3′ primers (the underlined bases indicate the T7 promoter) and cloned using the XhoI-MluI restriction sites in the pTRIPz-p53.

### Transfection and Lentiviral Transduction

Production of lentiviral supernatant was performed as previously described (29). Briefly, pTRIPZ shRNA anti-Chk1 constructs, pTRIPZ Inducible Lentiviral non-silencing shRNA Control (Cat n° RHS4743), pLEX p53R248Q-EGFP, pTRIPz-p53-EGFP transfer vectors and their respective empty vector controls were co-transfected with the packaging plasmids in the HEK293T cell line, using the calcium phosphate transfection method according to the Open Biosystem protocol (Trans-Lentiviral Packaging Kit, Open Biosystem). Viral supernatants were harvested 48 h after transfection.

The Namalwa cell line was then transduced with pTRIPz-EGFP or pTRIPz-p53-EGFP by a double round of spin-infection. Cells were centrifuged for 90 min at 32°C and 1,200 x g in the presence of 1 mL of viral supernatant and 8 μg/mL Polybrene (Sigma-Aldrich). Forty-eight hours post-transduction, Namalwa cells were finally selected with 1 μg/mL puromycin (Sigma-Aldrich) for 72 h.

BL-2 and SUP-B15 cell lines were transduced using 100-fold concentrated viral supernatants as previously described (30). Cells were subjected to two rounds of spinoculation as specified above and selected with 1 μg/mL puromycin for 72 h.

### Cell Proliferation and Cell Death Assay

To measure the IC_50_ of IO or Gemtuzumab Ozogamicin (GO), 1 × 10^4^ BL-2, SUP-B15, Namalwa, Ramos, Raji, HL-60 and lentivirally transduced BL-2 and SUP-B15 cells or 2.5 × 10^4^ primary leukemic cells were seeded in triplicate in 96-well plates and incubated for 48 h in the presence of increasing logarithmic doses of IO or GO (a gift of Pfizer) expressed as equivalents of N-acetyl-γ-calicheamicin dimethylhydrazide as previously reported (31). The doses employed ranged from 0.001 to 1000 ng/mL CalichDMH. Cell proliferation was determined using the luminescence ATP detection assay system ATPlite 1 step (Perkin-Elmer), following the manufacturer's instructions as previously reported (32). Subsequently, the IC_50_ value was calculated by logistic non-linear regression using Prism 5.0 Software (GraphPad Software Inc) and was reported as the amount of CalichDMH equivalents (nM) from each treatment group that caused 50% loss of cell viability.

To calculate IO IC_50_ for Namalwa cells transduced with pTRIPz-EGFP empty vector or pTRIPz-p53-EGFP, 1 × 10^4^ cells—either untreated or incubated with doxycycline (Sigma Aldrich) 1 μg/ml—were incubated with increasing logarithmic doses of IO (from 0.1 to 10.000 ng/mL CalichDMH) for 24 h. Cell viability and IC_50_ values were determined as previously described.

To estimate cell viability after p53 inhibition, 1 × 10^4^ Namalwa and Raji and 2 × 10^4^ Ramos cells were plated in triplicate in a 96-well plate and incubated for 24 h with their IO IC_50_ value alone or in combination with 20 μM pifithrin-alpha (Sigma—Aldrich) (Namalwa and Raji) or 2.66 μM APR-246 (PRIMA-1^MET^) (Selleckem) (Ramos). Cell proliferation was evaluated using an MTS assay (CellTiter 96® Aqueous One Solution Cell Proliferation Assay; Promega) following the manufacturer's instructions.

To analyze cell death, 1 × 10^4^ BL-2, SUP-B15, Namalwa and Raji and 2 × 10^4^ Ramos cells were treated with CalichDMH equivalents corresponding to their IO IC_50_, with 100 nM UCN-01 (Merk Biosciences) or p53 inhibitors, alone or in combination. Cells were then harvested and their apoptotic rate was determined using the Annexin V FITC/ 7AAD kit (Beckman Coulter) following the manufacturer's instructions.

The Namalwa cell line transduced with the non-silencing shRNA or with anti-Chk1 shRNAs was induced with 1 μg/ml doxycycline for 24 h before performing death assays. Cells were kept in the presence of doxycicline for an additional 24 h to maintain shRNA silencing and were then treated with IO. They were then harvested and apoptosis was measured using the Annexin V FITC/ 7AAD kit following the manufacturer's instructions.

For primary BM mononuclear samples, 20 × 10^4^ leukemic cells were plated on a stromal feeder and then exposed to IO or UCN-01, alone or in combination. Cells were initially stained with monoclonal antibodies anti-CD45-Phycoerythrin Cyanin 7 (PC7) (clone J33) and anti human CD19-R-Phycoerythrin-Texas Red (ECD) (clone J3-119) (both Beckman Coulter) and incubated in the dark for 20 min at room temperature, then washed in PBS and finally labeled with the Annexin V FITC/7AAD kit according to the manufacturer's instructions. All data acquisition and analysis were performed using a Cytomics FC500 flow-cytometer (Beckman Coulter).

### Immunoblotting

Cell pellets were resuspended in Laemmli buffer [62.5 mM Tris-HCl (pH 6.8), 2% w/v SDS, 10% glycerol, 50 mM DTT, 0.01% w/v bromophenol blue], sonicated, denatured for 5 min and separated on SDS-PAGE. The proteins were then transferred to nitrocellulose membranes and blocked with 5% non-fat dry milk or with 5% Bovine Serum Albumin (Sigma-Aldrich) in Tris-Buffered Saline with 0.1% Tween 20 (Sigma-Aldrich).

Primary antibodies used were: polyclonal anti-phosphoChk1 (Ser345), monoclonal anti-Chk1, polyclonal anti-phosphoChk2 (Thr68) and monoclonal anti-Chk2 from Cell Signaling; polyclonal anti-p21, monoclonal anti-p53, polyclonal anti-phosphoCdc25C (Ser216) and monoclonal Cdc25C from Santa Cruz Biotechnology; monoclonal anti-GFP (Covance); monoclonal anti-Actin (Sigma-Aldrich). Appropriate horseradish peroxidase conjugated secondary antibodies (Amersham Biosciences) were added and proteins were then detected using the enhanced chemiluminescence reagent ECL Star (Euroclone).

### Cell Cycle Distribution Analysis

1 × 10^4^ BL-2, SUP-B15 and Namalwa cells were plated and treated with their respective IO IC_50_ for 12, 24 and 48 h. Cells were then harvested, fixed in 70% of ethanol in Phosphate-Buffered Saline (PBS) for 24 h at −20°C and incubated with 40 μg/mL RNase A and 20 μg/mL Propidium Iodide (both from Sigma-Aldrich) for 30 min. Cell cycle distribution was then evaluated employing the Cell Quest software (Becton-Dickinson) for acquisition and WinMDI 2.9 Software (Joseph Trotter, The Scripps Institute, La Jolla, CA) and Cylchred Software packages (Cell Cycle Analysis Software, Cardiff, UK) for analysis.

### Statistical Analysis

Statistical analysis was performed using GraphPad Prism 5.0a (GraphPad Software Inc). Unpaired, single-tail *t*-tests with 95% confidence intervals were used to compare cell viability in different experimental conditions. The 1-way ANOVAs according to Bonferroni's post-test were used to compare the effect of IO on cell cycle phases at different time points.

## Results

### Inotuzumab Ozogamicin Shows Different Anti-proliferative Efficacy on CD22-Positive Leukemic Cell Lines With Different p53 Status

To ascertain if the CD22-specific cytotoxicity of IO would be influenced by p53 expression, we employed three cell lines chosen for their diverse p53 profiles as BL-2 cells express wild-type p53, SUP-B15 present low levels of wild-type p53 (because of *MDM*2 gene amplification) and Namalwa cells display the p53 mutation R248Q (33).

**Figure 1:**
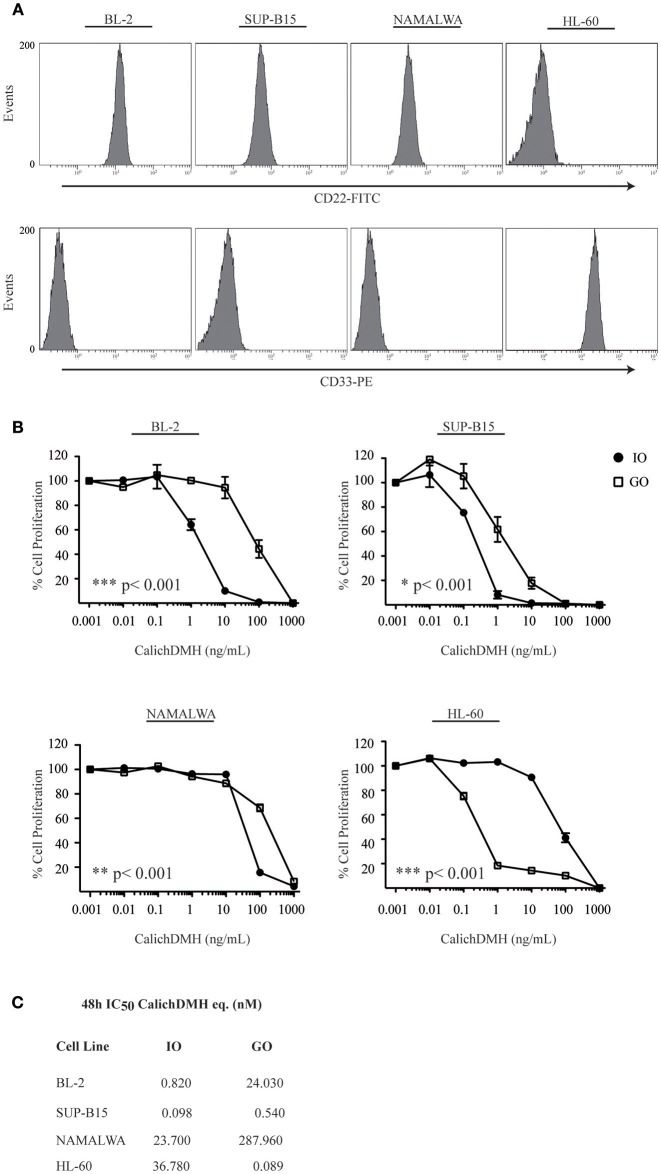
Antiproliferative effect of Inotuzumab Ozogamicin and Gemtuzumab Ozogamicin on human malignant CD22-positive cell lines. **(A)** Relative expression of CD22 and CD33 antigens on the cell surface of BL-2, SUP-B15, Namalwa and HL-60 cells. **(B)** Reductions in cell proliferation rates were calculated for CD22-positive/CD33-negative (BL-2, SUP-B15 and Namalwa) and CD22-negative/CD33-positive (HL-60) cell lines after a 48 h incubation with increasing concentrations of anti-CD22 (IO, •) and anti-CD33 (GO, □) calicheamicin immunoconjugates. Results represent the average ± standard deviation of at least three different experiments performed in triplicates with relative luminescence of untreated cells arbitrarily set at 100%. **(C)** IC_50_ values were calculated by logistic non-linear regression and are presented in the table as nM equivalents of CalichDMH for IO and GO, respectively.

FACS analysis confirmed expression of the CD22 B-lymphoid antigen in >95% of BL-2, SUP-B15 and Namalwa cells while the myeloid-specific CD33 antigen was not detected in any of the above-mentioned cells but was expressed in 94% of the Acute Myeloid Leukemia cell line HL-60 employed as a negative control (Figure 1A).

We then incubated the above-mentioned cell lines with increasing doses of CalichDMH-conjugates (IO or the anti-CD33 ADC Gemtuzumab Ozogamicin-GO) and found that IO was much more effective than GO in reducing cell proliferation (Figure 1B), generating IC_50_ values that were consistently lower than those of their anti-CD33 counterpart (Figure 1C). As expected, in CD33-positive HL-60 cells, GO was >400 fold more effective than IO. BL-2 and SUP-B15 were very sensitive to IO displaying IC_50_ values (expressed as nM of CalichDMH equivalents) of 0.82 and 0.098, respectively. On the contrary, the Namalwa cell line was considerably less responsive to the drug, displaying an IC_50_ of 23.70 nM that was comparable to that observed in the CD22-negative HL-60 cells (36.78 nM).

### Inotuzumab Ozogamicin Induces a G2/M Arrest That Is Associated With Chk1 and Chk2 Phosphorylation

**Figure 2:**
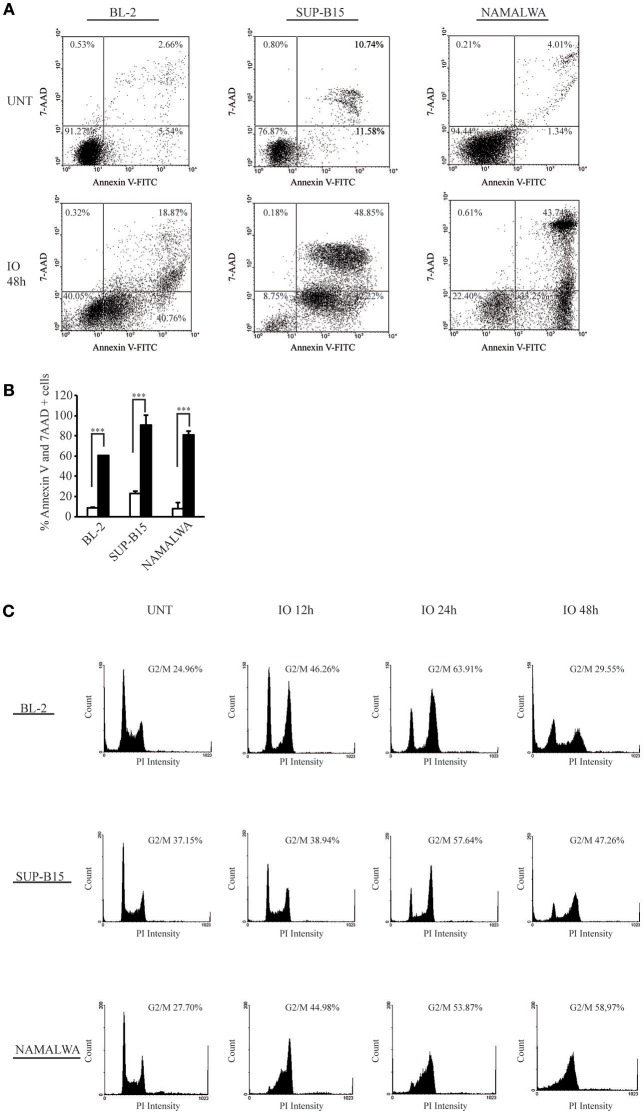
CD22-expressing cells that survive Inotuzumab Ozogamicin are blocked in the G2/M phase of the cell cycle. **(A)** Representative dot plots showing the apoptotic rates observed in BL-2, SUP-B15 and Namalwa cells. The indicated CD22-positive cell lines were either left untreated (UNT) or treated with calicheamicin equivalents corresponding to their IO IC_50_ values for 48 h. Apoptotic rates were evaluated using flow cytometry after Annexin V-FITC/7AAD double staining. The indicated percentage values show the distribution of viable and necrotic/apoptotic cells for each condition. **(B)** Histograms representing the average percentage of Annexin V and 7 AAD positive cells in the untreated (white columns) or IO treated (with their respective IC_50_ values; black columns) conditions. Columns represent average ± standard deviation of three independent experiments performed in triplicates. ^***^p < 0.001 **(C)** Representative experiment displaying the cell cycle distribution of BL-2, SUP-B15 and Namalwa cells. Each line was treated for 12, 24, and 48 h with it's respective IO IC_50_ value and surviving cells were then analyzed for cell cycle distribution by flow cytometry using propidium iodide (PI). The percentage of cells blocked in G2/M is indicated for each panel.

Next, we wanted to determine if the reduced proliferation rate that we observed in CD22-positive cells after IO treatment was due to induction of programmed cell death or to cell-cycle arrest. To this end, we incubated BL-2, SUP-B15 and Namalwa cells for 48 h using IO concentrations reflecting their IC_50_ values and then measured cell death by staining cells with Annexin V/7AAD (Figures 2A,B). We found that IO killed 60% of BL-2, 91% of SUP-B15 and 81% of Namalwa cell lines compared to 9, 23, and 8% of apoptotic rates in the untreated condition (Figures 2A,B). While these apoptotic rates may seem comparable, the fact that Namalwa cells required IO doses 29 fold (BL-2) and 241 fold (SUP-B15) higher than those employed for the other CD22-positive cell lines suggested a possible CD22-independent cytotoxicity derived from the release of unbound CalichDMH as previously described (31).

We then analyzed the cell cycle progression of each cell line after IO exposure for 12, 24, and 48 h. We found that cells surviving drug treatment exhibited a modified cell-cycle profile as both BL-2 and SUP-B15 displayed a progressive increase in the population blocked in the G2/M phase of the cell cycle (Figure 2C and Table 1). Specifically, BL-2 cells exhibited a G2/M arrest in 46.26% of the population after 12 h of drug exposure that increased to 65.40% after 24 h. Likewise, the SUP-B15 cell line presented 38.94% of cells in G2/M after 12 h of IO with a further increase to 57.64% after 24 h. Both in BL-2 and SUP-B15, we detected a decrease of the G2/M population after 48 h of IO incubation (29.55% for BL-2 and 47.26% for SUP-B15), due to the increase in the subG0 population killed by the drug. On the contrary, IO treatment of the Namalwa cell line determined an early block in G2/M (44.98% at 12 h) that was maintained even 24 (53.87%) and 48 h (58.97%) after treatment (Table 1 and Figure 2C).

**Table 1:** Cell cycle distribution of BL-2, SUP-B15, and Namalwa cells in untreated and Inotuzumab Ozogamicin treated samples at different time points.

**Cell line**	**Group**	**Cell cycle distribution**			
		**subG1**	**G1**	**S**	**G2/M**
BL-2	UNT	12.70 ± 0.91	43.46 ± 2.82	19.13 ± 0.96	24.70 ± 1.63
	IO 12 h	2.66 ± 0.15^***^	37.25 ± 1.67^*^	13.84 ± 0.90^***^	46.26 ± 2.31^***^
	IO 24 h	3.85 ± 0.14^***^	24.18 ± 1.57^***^	5.75 ± 0.41^***^	65.40 ± 5.58^***^
	IO 48 h	23.96 ± 1.08^***^	30.78 ± 3.10^***^	14.56 ± 0.82^***^	29.94 ± 2.10^ns^
SUP-B15	UNT	4.85 ± 0.19	40.46 ± 3.09	16.40 ± 0.91	37.70 ± 2.29
	IO 12 h	5.64 ± 0.27^ns^	32.27 ± 1.82^**^	22.81 ± 1.95^***^	39.59 ± 2.38^ns^
	IO 24 h	5.98 ± 0.19^ns^	19.37 ± 0.79^***^	15.56 ± 1.12^ns^	58.45 ± 2.64^***^
	IO 48 h	18.30 ± 1.97^***^	17.27 ± 0.74^***^	15.55 ± 1.05^ns^	48.02 ± 2.47^**^
NAMALWA	UNT	3.91 ± 0.17	43.30 ± 2.87	21.63 ± 1.06	30.96 ± 1.21
	IO 12 h	2.70 ± 0.15^**^	6.70 ± 0.28^***^	44.41 ± 2.22^***^	45.79 ± 2.75^**^
	IO 24 h	5.89 ± 0.26^***^	12.67 ± 0.46^***^	26.10 ± 1.82^*^	55.51 ± 3.44^***^
	IO 48 h	9.45 ± 0.62^***^	13.63 ± 0.67^***^	16.72 ± 1.17^*^	59.07 ± 4.42^***^

Statistical analysis indicates significance on variation in percentage of cell cycle distribution after treatment with IO at different time points compared to the untreated (UNT) condition. Results are expressed as mean ± standard deviation values from three independent experiments.

* p < 0.01;

** p < 0.01;

*** *p < 0.001; ns, not significant*.

Our results confirm that IO arrests cells in the G2/M phase of the cell cycle (16), but indicate that this block is differently modulated in BL-2 and SUP-B15 cells as compared to the Namalwa cell line.

**Figure 3:**
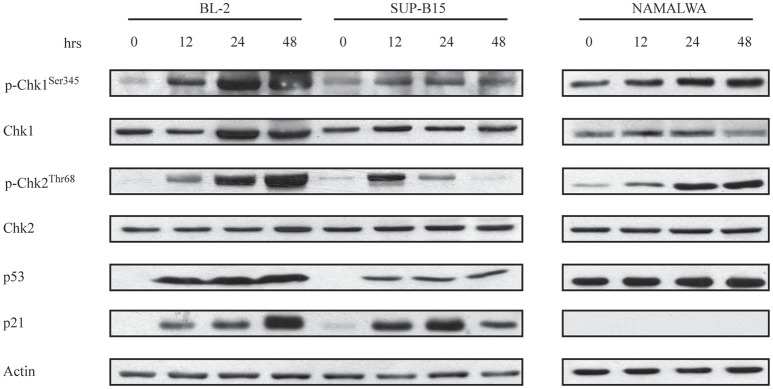
Chk1 and Chk2 are involved in the G2/M arrest induced by Inotuzumab Ozogamicin. BL-2, SUP-B15 and Namalwa cell lines were exposed to IO according to their IC_50_ values, for the indicated time points. Cells were then lysed and protein extracts were used to perform immunoblots employing the specified antibodies. Actin was used as a loading control. The depicted blots are representative of three separate experiments.

Given these findings, we wanted to establish if the differing p53 status of our CD22-positive cell lines contributed to their different response to IO. Consolidated evidence has shown that induction of DNA damage can block cell cycle progression by triggering multiple signal transduction hubs (34, 35) that converge on the Chk1/Chk2/p53/p21 pathway (36). To establish if this was the case for the G2/M arrest displayed by CD22-positive cells exposed to IO, we analyzed their protein lysates after drug incubation for 12, 24, and 48 h and found that all cells displayed a considerable increase in Chk1 phosphorylation (Figure 3). Moreover, BL-2 and Namalwa also exhibited increased Chk2 phosphorylation at each considered time point. As Chk1 and Chk2 induction by DNA damage results in p53 activation (37, 38), we investigated p53 expression after IO exposure and detected its up-regulation in BL-2 and SUP-B15 cells. As expected, we failed to observe variations in the p53 levels of Namalwa cells as they overexpress mutant p53 at baseline (Figure 3). To confirm preservation of p53-dependent transcriptional activity, we analyzed p21 protein expression and detected p21 induction in both BL-2 and SUP-B15 but not in the Namalwa line (Figure 3).

Taken together these findings indicate that IO-dependent G2/M cell cycle arrest is associated with Chk1 phosphorylation. Our results also suggest that successful IO-mediated killing may require wild-type p53 as Namalwa cells were poorly responsive to IO despite their high CD22 expression.

### The Sequential Combination of Inotuzumab Ozogamicin and the Chk1 Inhibitor UCN-01 Increases the Apoptotic Rate of CD22-Positive Leukemic Cells

**Figure 4:**
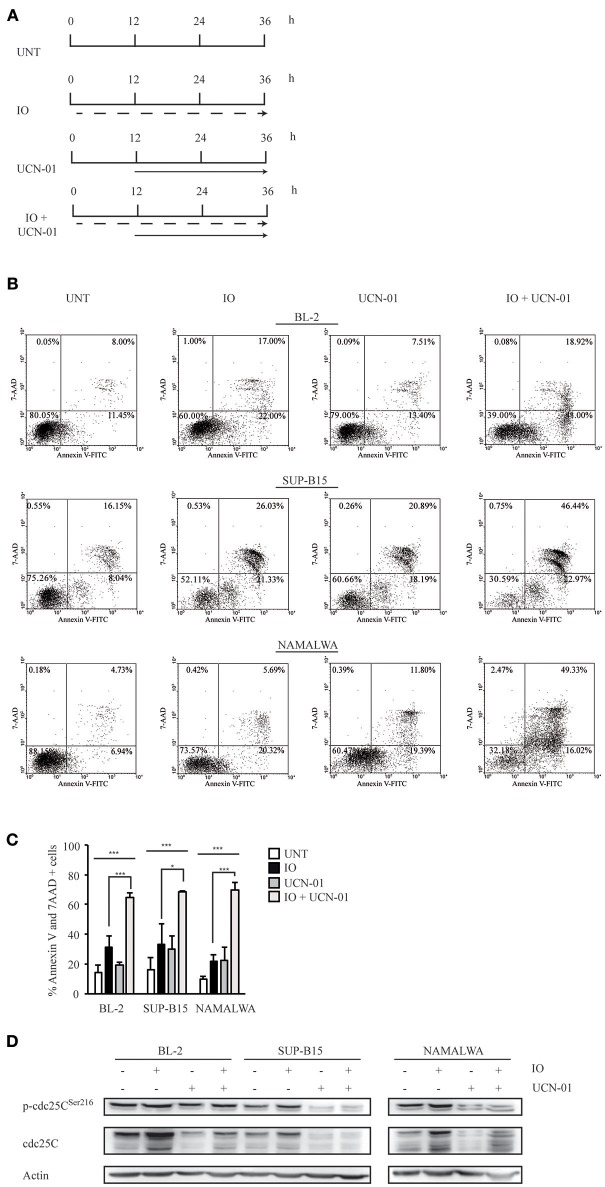
Treatment with Inotuzumab Ozogamicin and UCN-01 determines an increase in the apoptotic rate of CD22-positive cells. **(A)** Treatment scheme employed in the experiments described in panels (B–D). BL-2, SUP-B15 and Namalwa cell lines were either left untreated (UNT) or were exposed to IO (dashed arrow) according to their respective IC_50_ values, to 100 nM UCN-01 (solid arrow) or to a combination of the two drugs. In the latter case cell lines were kept for 12 h in IO and UCN-01 was added for the remaining 24 h of the experiment. **(B)** Representative experiment displaying the apoptotic rates detected in BL-2, SUP-B15 and Namalwa cell lines. The indicated cells were grown in the absence of drugs or treated with IO or UCN-01. Apoptosis was then evaluated after Annexin V-FITC/7AAD double staining. The indicated percentages show the distribution of necrotic, early and late apoptotic cells after IO and UCN-01 treatment, alone or in combination. **(C)** Histograms representing the average percentage of Annexin V and 7 AAD-positive cells in the untreated condition or after exposure to IO, UCN-01 or a combination of the two drugs. Columns represent average ± standard deviation of three independent experiments performed in triplicates. ^*^p < 0.05, ^***^p < 0.001 **(D)** Cell lines treated, as specified in (A), were also harvested, lysed and probed with the specified antibodies to perform immunoblot assays. Actin was used as a loading control. The depicted blots are representative of three separate experiments.

Several reports have indicated that Chk1 pharmacological inhibition by UCN-01 increases the cytotoxic effect of different chemotherapeutic agents by abrogating Chk1-induced cell cycle arrest via both p53-dependent and -independent mechanisms (39–41). To establish if we could increase the IO sensitivity of Namalwa cells, we employed the sequential combination of IO and UCN-01 as depicted in Figure 4A. We found that, in BL-2 and SUP-B15 cells displaying wild-type p53, the two-drug combination increased apoptosis by 1.5 fold (Figures 4B,C). Strikingly, when we repeated this experiment on the Namalwa cell line employing an IO concentration calculated by averaging the IC_50_ values of BL-2 and SUP-B15 responsive cells (0.459 nM), we observed a 2.6 fold increase in cell death as compared to IO alone (67.82% vs 26.43%) (Figures 4B,C). Furthermore, this result was achieved employing IO concentrations that were 50 fold lower than the previously calculated IC_50_ for Namalwa cells (Figure 1C).

As UCN-01 is a staurosporine analog displaying multiple targets we could not exclude a non-specific effect attributable to the many substrates of this drug. We therefore silenced Chk1 expression in the Namalwa cell line employing a pool of inducible anti-Chk1 shRNAs. We initially performed an immunoblot to verify Chk1 silencing after 24 and 48 h of doxycycline induction (Supplemental Figure 1A). We next measured the apoptotic rate of cells transduced with the control non-silencing shRNA (shRNA NS) or with the anti-Chk1 specific shRNA and treated for 24 h with an IO concentration calculated by averaging the IC_50_ values of BL-2 and SUP-B15 responsive cells.

The silencing of Chk1 expression and the treatment with IO produced a 1.8 fold increase in cell death as compared to IO alone obtained after shRNA NS induction (29.83 vs 16.72%) (Supplemental Figures 1B,C).

While these data implied the presence of a strong Chk1-dependent increase in cell death, we still had not defined which protein downstream of Chk1 contributed to this event. It is well-established that—after DNA damage—activated Chk1 phosphorylates the cdc25C phosphatase, thereby favoring its interaction with 14-3-3 proteins that results in the nuclear export of cdc25C. Cytoplasmic retention by 14-3-3 prevents mitosis progression thus determining a G2/M arrest (42). To verify if UCN-01-dependent inhibition of Chk1 by-passed IO-induced cell cycle arrest, we performed an anti-phospho-cdc25C immunoblot. We found that UCN-01—alone or in combination with IO—reduced both cdc25C expression and phosphorylation (Figure 4D).

These results suggest that inhibition of Chk1 activity by UCN-01 may abolish IO-dependent G2/M arrest, inducing significant increases in cell death regardless of the p53 cellular background.

### p53 Status Determines Inotuzumab Ozogamicin Efficacy on Immortalized CD22-Positive Leukemic Cells

To provide genetic confirmation that p53 status determines IO sensitivity in CD22-positive cells, we introduced the R248Q mutant p53 in cells harboring wild-type p53 and - conversely - expressed wild-type p53 in the Namalwa cell line.

**Figure 5:**
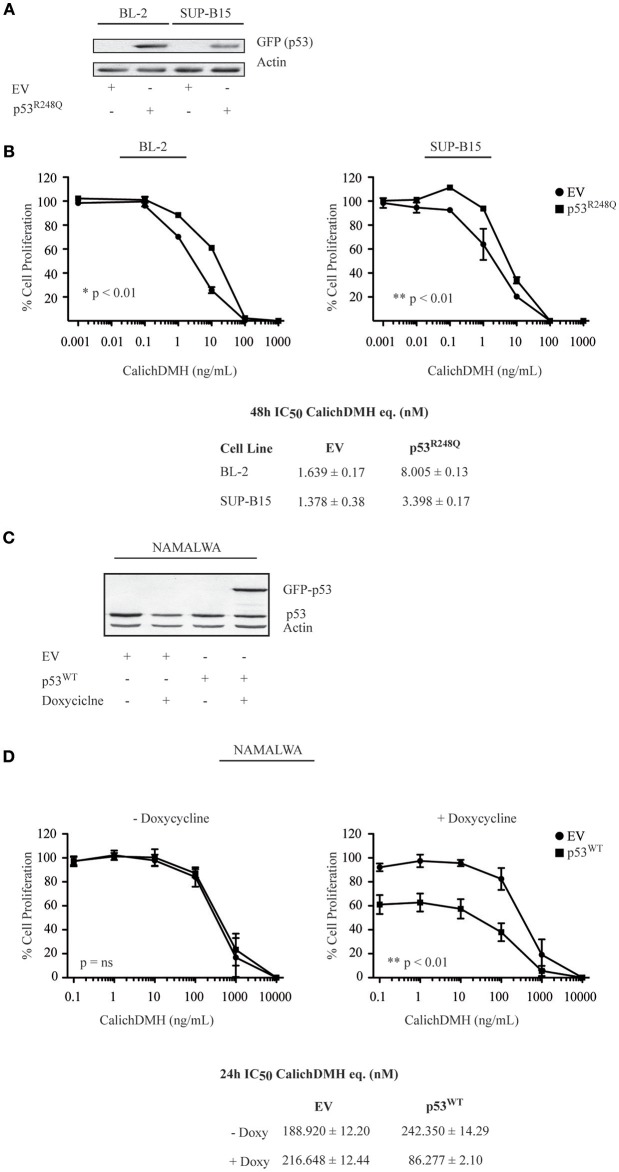
p53 wild-type increases the sensitivity of CD22-positive cells to Inotuzumab Ozogamicin. **(A)** BL-2 and SUP-B15 cell lines were lentivirally transduced with an empty vector (EV) or the GFP-tagged p53^R248Q^-pLEX construct. After puromycin selection, cells were lysed and protein extracts were blotted using an anti-GFP antibody to verify protein expression. Actin was employed as a loading control. **(B)** The modified cell lines, stably expressing either EV (•) or mutant p53 (■) were then exposed to logarithmic doses of IO and their reduction in cell proliferation was evaluated employing the ATPLite luminescence assay. Results represent the average ± standard deviation of at least three different experiments performed in triplicates with relative luminescence of untreated cells arbitrarily set at 100%. IC_50_ values were calculated by logistic non-linear regression and are presented as nM equivalents of CalichDMH. **(C)** Namalwa cells were transduced with an empty vector (EV) or GFP-tagged wild-type (WT) p53—pTRIPZ vector using an inducible lentiviral system. After puromycin selection, cells were treated for 24 h with doxycycline to induce wild-type p53 expression. Cells were harvested, lysed and protein extracts were simultaneously blotted for p53 and Actin, the latter employed as a loading control. **(D)** The reductions in proliferation of Namalwa cells expressing EV (•) or p53^WT^ (■) were then evaluated after co-treatment for 24 h with doxycycline and logarithmic doses of CalichDMH. Results represent the average ± standard deviation of at least three different experiments performed in triplicates with relative luminescence of untreated cells set arbitrarily at 100%. IC_50_ values are reported as nM equivalents of CalichDMH.

Specifically, BL-2 and SUP-B15 were stably transduced with lentiviral vectors expressing either GFP-tagged p53^R248Q^ or an empty vector used as a control. Anti-GFP immunoblots confirmed expression of the p53^R248Q^ mutant (Figure 5A). Transduced cells were subsequently exposed to different IO concentrations to determine their 48-h IC_50_. We observed that cells overexpressing p53^R248Q^ presented higher IC_50_ values than the empty vector-transduced counterpart, requiring a 5-fold (BL-2: from 1.63 to 8 nM) or a 2.5-fold increase (SUP-B15: from 1.37 to 3.4 nM) to achieve 50% cell killing (Figure 5B). Since expression of wild-type p53 induces a marked cytotoxic effect, we engineered a doxycycline-inducible lentiviral vector expressing the EGFP-tagged p53 and used it to transduce the Namalwa cell line. An immunoblot confirmed that doxycycline exposure for 24 h induced wild-type p53 expression (Figure 5C). We then exposed the transduced Namalwa cells to increasing IO concentrations in the presence of doxycycline. As p53 induction *per se* determines an increase in cell death, we decreased the time of exposure to IO and used drug doses ranging from 0.1 to 10.000 ng/mL CalichDMH. When we determined the cells IC_50_, we observed different values as compared to those initially calculated for Namalwa cells and indicated in Figure 1. This finding was not surprising as the cells were continuously cultivated for 8 weeks (to allow lentiviral transduction and puromycin selection) and were incubated with IO for a different time (24 vs. 48 h) than the one employed in our previous experiments.

Moreover, we found that forcing wild-type p53 expression in Namalwa cells increased anti-CD22 CalichDMH sensitivity, recording an IC_50_ value 2.5 fold lower than that displayed by the empty vector-induced cells (86.27 vs. 216.64 nM). On the contrary, minimal differences were observed in the IC_50_ values of cells that were not incubated with doxycycline (i.e., no p53 induction) or were transduced with the empty vector control (Figure 5D).

To further validate the role of mutant p53 in determining a reduced IO sensitivity in CD22-positive cells, we inhibited p53 expression in Namalwa cells and in two other lines expressing mutant p53 isoforms. To this end, we employed Raji cells that express the R273H gain of function mutation that involve an amino acidic residue directly involved in DNA binding and is therefore devoid of transcriptional activity (43) and the Ramos cell line displaying the I254D loss of function mutation (44).

To confirm the importance of wild-type p53 in contributing to different responses to IO we calculated the drug's IC_50_ in Raji and Ramos cell lines. As expected, after 48 h of treatment we found Raji and Ramos cells to be poorly responsive to IO displaying IC_50_ values of 54.56 and 185.5 nM, respectively (data not shown).

**Figure 6:**
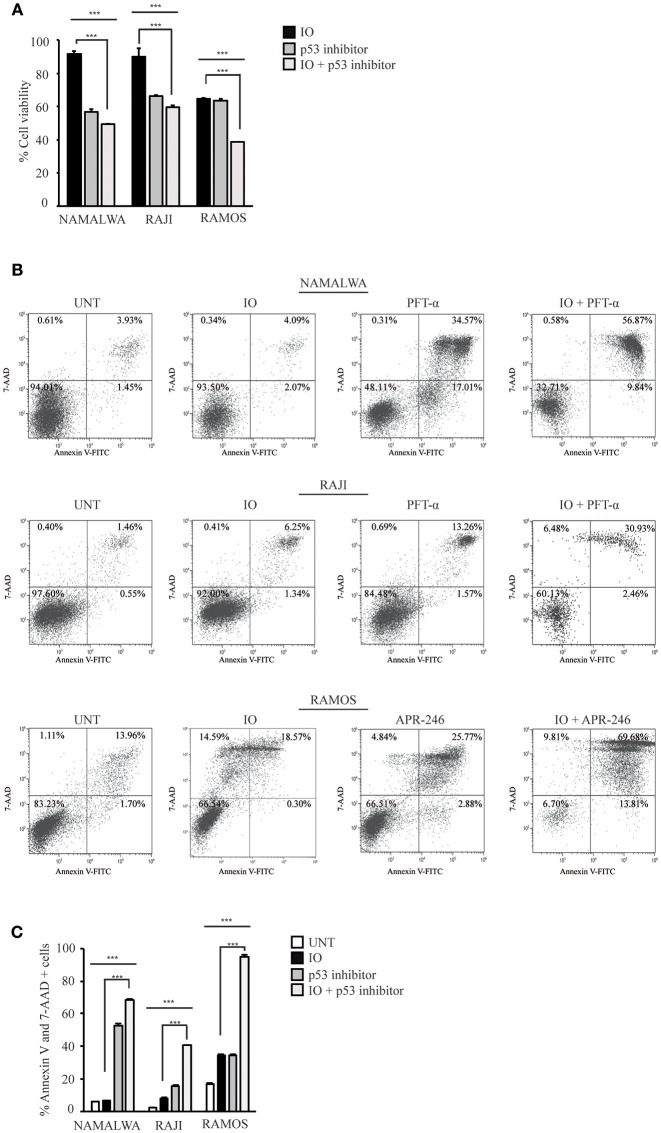
Inhibition of mutant p53 increases the sensitivity of CD22-positive cells to Inotuzumab Ozogamicin. **(A)** Namalwa, Raji and Ramos cell lines expressing mutant p53 were grown in the absence of drugs or treated with their IC_50_ IO values and p53 inhibitors alone or in combinations for 24 h. Namalwa and Raji were treated with 20 μM pifitrhin-alpha (PFT-α) while Ramos cells were treated with their APR-246 (PRIMA-1^Met^) IC_50_ value. Cell lines were then subjected to MTS proliferation assays. Histograms show relative percentage of metabolically active cells with the untreated condition arbitrarily set at 100%. Columns represent average ± standard deviation of two independent experiments carried out in triplicates. **(B)** Representative experiment on the indicated CD22-positive cell lines either left untreated (UNT) or treated as described in panel A. Apoptotic rates were then evaluated using flow cytometry after Annexin V-FITC/7AAD double staining. The indicated percentage values indicate the distribution of viable and necrotic/apoptotic cells for each condition. **(C)** Histograms representing the average percentage of Annexin V and 7 AAD-positive cells in the untreated condition or after exposure to IO, pifithrin-alpha or APR-246 or a combination of these drugs. Columns represent average ± standard deviation of three independent experiments performed in triplicates. ^***^p < 0.001.

Several reports have indicated that chemical inhibition of p53 gain of function mutations by pifithrin-alpha (PFT-α) (45) or restoration of a p53 active conformation with APR-246 (also named PRIMA-1^Met^) (46) increases the apoptosis of cells expressing different mutant p53 isoforms. To establish if IO sensitivity is dependent on p53 status, we co-treated Namalwa and Raji cell lines with IO and PFT-α or alternatively exposed Ramos cells to the combination of IO and APR-246. When we assayed cell proliferation and survival in the presence of IO and PFT-α or APR-246 we found significant reduction on cell growth and viability of all p53 mutated cell lines. Specifically, we found that co-treatment with IO and PFT-α or APR-246 for 24 h caused a decrease in cell viability of about 1.7 fold compared to treatment with IO alone in all three cell lines (Figure 6A). Furthermore, we observed an increase in the amount of apoptotic cells from 6.7 to 68.3% in Namalwa, from 8.2 to 40.5% in Raji and from 34.3 to 94.7% in Ramos cells (Figures 6B,C).

Our data suggest that the anti-proliferative effect of IO is heavily influenced by p53 status, suggesting that p53 evaluation may predict the response of CD22-positive leukemic cells to a Calicheamicin derivative.

### p53 Modulates Inotuzumab Ozogamicin Efficacy on Primary CD22-Positive Leukemic Cells

**Figure 7:**
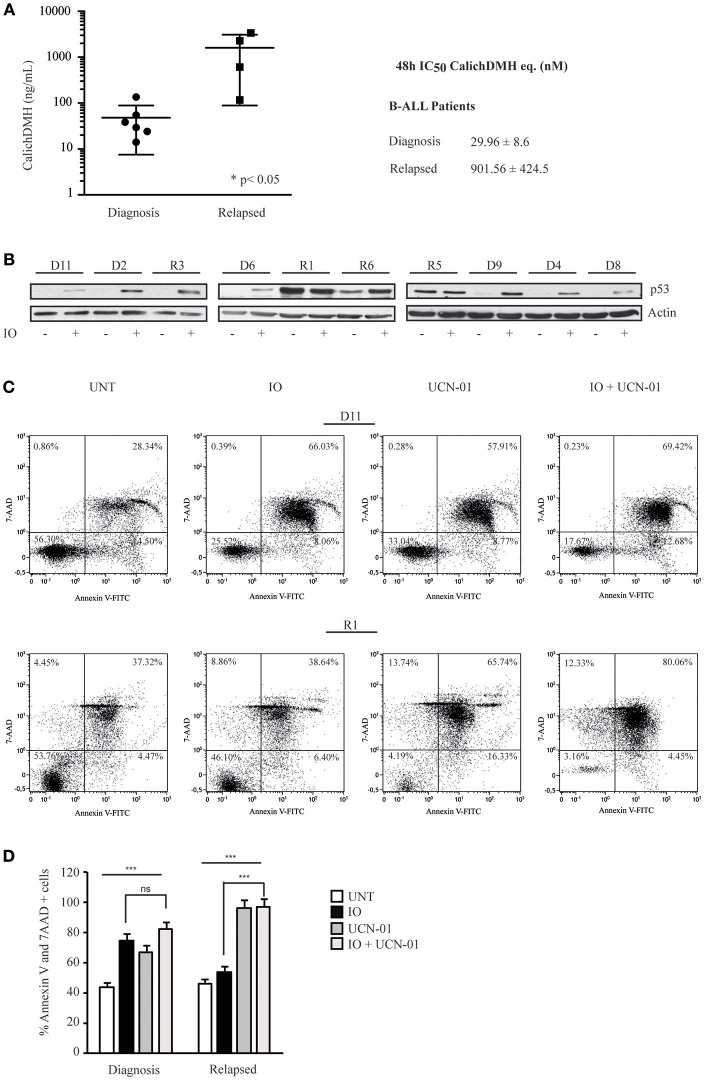
Primary human CD22-positive cells expressing wild-type p53 display high sensitivity to Inotuzumab Ozogamicin. **(A)** Reduction in cell viability calculated on mononuclear cells derived from six CD22-positive human B-ALL patients at diagnosis and four patients with r/r B-ALL. After a 48 h incubation with increasing IO concentrations, the reduction of cell proliferation was evaluated employing the ATPLite luminescence assay. The right panel indicates average ± standard deviation of the IC_50_ values, expressed as nM equivalents of CalichDMH. **(B)** The indicated primary cells derived from B-ALL patients at diagnosis (D) or from r/r B-ALL (R) were also treated for 48 h with calicheamicin equivalents corresponding to their IO IC_50_ average values. Lysates from these cells were then blotted using the specified antibodies. Actin was used as a loading control. **(C)** Primary cells, derived from one B-ALL (D11) and one r/r B-ALL (R1) patient, were left untreated or exposed to the IO IC_50_ average value for B-ALL patients at diagnosis (29.96 nM) and UCN-01 100 nM, alone or in combination, employing the scheme described in Figure 4A. Apoptosis was then evaluated after Annexin V-FITC/7AAD double staining. The indicated percentage values show the distribution of necrotic, early and late apoptotic cells. **(D)** Histograms representing the average percentage of Annexin V and 7 AAD positive cells in the untreated condition or after exposure to IO, UCN-01 or a combination of the two drugs. Columns represent average ± standard deviation of two independent experiments. ^***^p < 0.001; ns, not significant.

To validate the data generated in immortalized cell lines, we isolated mononuclear cells from six newly diagnosed B-ALL patients and four subjects presenting refractory or relapsed B-ALL (r/r B-ALL). These cells were incubated with logarithmic IO concentrations for 48 h and evaluated for cell viability (Figure 7A). Our experiment showed that cells derived from newly diagnosed B-ALL were more sensitive to IO than r/r B-ALL cells, with the former displaying average IC_50_ values of 29.96 nM and the latter presenting an average IC_50_ of 901.56 nM (Figure 7A).

While alterations in p53 are considered an infrequent event in B-ALL, the disruption of both p53 alleles or the presence of missense mutations is associated with an adverse prognosis (47, 48). To further confirm that the higher IC_50_ values displayed by r/r B-ALL patients correlated with their p53 status, we initially analyzed their p53 expression levels after a 48 hour incubation with IO. As reported in Figure 7B, we found that newly diagnosed B-ALL patients only expressed p53 after IO treatment, while three (R1, R5, and R6) of the four r/r B-ALL subjects in our series displayed p53 overexpression in the untreated condition suggesting the presence of a mutant p53. Indeed, the r/r B-ALL patients who exhibited the highest IC_50_ values also showed a mutant p53.

We next wanted to establish if Chk1 inhibition would increase IO efficacy on primary B-lymphoid cells. Hence, these cells were exposed to the IO and UCN-01 combination previously described and scored for cell death. When we incubated cells derived from a B-ALL patient at diagnosis with 29.96 nM IO (mean of the IC_50_ values of B-ALL patients at diagnosis) and UCN-01—alone or in combination—we detected no significant differences in the amount of cell death between IO plus UCN-01 (82.33%) and IO alone (74.48%; Figures 7C,D). However, when we repeated this experiment using primary cells derived from a r/r B-ALL patient expressing mutant p53, addition of UCN-01 to IO doubled the apoptotic rate compared to IO alone (from 53.9 to 96.84%) employing an IO dose 30-fold lower than the mean IC_50_ calculated for r/r B-ALL patients (Figures 7C,D). Furthermore, we observed remarkable cell death rates after treatment with UCN-01 alone, confirming the importance of Chk1 inhibition in B-ALL patients (49).

These findings strengthen our hypothesis that Chk1 phosphorylation triggers a G2/M arrest that will evolve in apoptosis in the presence of wild-type p53. Our data also suggest that p53 mutations may represent a limiting factor negatively modulating IO efficacy in patients with CD22-positive lymphoproliferative disorders.

## Discussion

While antibody-drug conjugates represent one of the many successful therapeutic strategies recently introduced in the fight against cancer, selected neoplastic clones eventually escape the cell killing mechanisms induced by these drugs (50). To explain the resistance to IO we investigated the intracellular signaling triggered by the drug and report several findings with potential clinical consequences for patients diagnosed with B-cell derived disorders receiving IO.

First of all, while it has been previously reported that CD22 levels play a minor role in determining IO efficacy, the general consensus is that high CD22 expression accelerates IO-induced cell death (51). Our data contradict this conclusion as we found that both immortalized cell lines (BL-2, SUP-B15, Namalwa) and primary cells exhibiting comparable CD22 levels displayed very different IO sensitivity. Hence, evaluation of CD22 expression cannot be considered a reliable biomarker to predict IO efficacy on CD22-positive cells.

We have also actively investigated the signaling network elicited by IO. As calicheamicin is a well-established DNA-damaging agent (13, 14), exposure to the compound would be expected to activate the ATM/ATR proteins (17) that, in turn, trigger the Chk1/Chk2/p38 pathway (52). This signaling network induces a reversible cell cycle arrest mediated by p53-dependent induction of p21 (53). This complex response to DNA damage represents an evolutionary selected failsafe mechanism aimed at preserving the DNA integrity of healthy cells (54, 55). Indeed, upon complete and accurate repair of the accumulated damage, cells may exit their cell cycle arrest and return to proliferate. However, in the presence of persisting (unrepaired or irreparable) DNA damage, cells will either senesce or undergo apoptosis (56).

Our data confirm that IO blocks cells in the G2/M phase of the cell cycle as previously described (16, 57). Moreover, the fact that this arrest was detected throughout our panel of immortalized cell lines and primary cells indicates that this event is independent of p53 status. However, we noticed that the initial G2/M block detected in all CD22-positive cells evolved in two alternative scenarios depending on their underlying p53. Indeed, while BL-2 and SUP-B15 (p53 wild-type) cells underwent apoptosis—possibly due to partial or inaccurate repair of IO-induced DNA-damage, p53-mutant Namalwa cells stabilized their G2/M arrest. Although we are still investigating this phenomenon, it is tempting to speculate that a prolonged G2/M arrest may enable cells to further repair their DNA damage, potentially favoring an escape from senescence by selected IO-resistant clones.

Our experiments also showed that, after IO exposure, the amount of death triggered by IO paralleled the p53 profile of the cells. Indeed, BL-2 and SUP-B15 expressing wild-type p53 both displayed high death rates (ranging between 60 and 90%) in the presence of <1 nM of CalichDMH equivalents. On the contrary, to achieve a 73% apoptotic rate, Namalwa cells with mutant p53 had to be incubated with large amounts of IO suggestive of cellular cytotoxicity derived from the release of antibody-free calicheamicin. Moreover, the IO IC_50_ for two further cell lines devoid a functional p53 confirmed that the CD22-specific cytotoxicity of IO is influenced by p53 expression. While these findings all point to p53 as a potential biomarker for IO efficacy, they are not in agreement with a previous report by Prokop and colleagues suggesting that p53 status bears no consequence on the apoptotic response triggered by calicheamicin (58). To explain these discrepancies, we engineered an inducible lentiviral vector expressing GFP-tagged p53 that was used to transduce Namalwa cells. In agreement with our previous observations, we found increased IO sensitivity (i.e., lower IC_50_) in the presence of wild-type p53. Furthermore, when we inhibited p53 transactivation (with PFT-α) or reactivated p53 function (with APR-246) in cell lines (Raji and Ramos) expressing gain or loss of function p53 mutations we again observed an increased sensitivity to IO. While we are devoid of a specific explanation for the discordance between our results and those previously published by Prokop and colleagues, it is possible to assume that the different cellular background between the two studies (colon cancer cells in the paper by Prokop et al. vs Burkitt's lymphoma and ALL cells in our study) or the use of the unconjugated drug (i.e., free calicheamicin unbound to the anti-CD22 antibody epratuzumab in the Prokop study) may have significantly contributed to these diverging results. We should also point out that the IO IC_50_ value calculated in primary cells derived from ALL patients at diagnosis or at the time of relapse also correlated with their p53 status, further strengthening the suggestion that p53 integrity predicts for IO efficacy.

Hence, our study confirms the promising results of a CD22-specific immunoconjugate in patients affected by B-cell lymphoproliferative disorders expressing wild-type p53. We also demonstrated that the combination of IO and the Chk1 inhibitor UCN-01 as well as Chk1 silencing forced G2/M-arrested cells to progress along the cell cycle increasing their apoptotic rate. Unexpectedly, we found that—unlike what we observed in immortalized cell lines—in B-ALL patients at diagnosis the combination of IO and UCN-01 did not determine a significant increase in cell death compared to IO alone. As it has been reported that the genomic profiling of B-ALL at diagnosis and relapse shows substantial changes in both the number and nature of the detected genetic alterations (59), it is possible to speculate that patients expressing wild-type p53 at diagnosis and lacking additional alterations in checkpoint pathways, remain sensitive to a DNA damaging agent but will not benefit from a checkpoint inhibitor (41). Our findings are in line with multiple studies suggesting the efficacy of Chk1 inhibitors as a new therapeutic strategy for B- and T-ALLs (49, 60). Furthermore, as in primary cells UCN-01 sensitivity is strictly related to the integrity of the p53 sequence, we hypothesize that the combination of IO and a Chk1 inhibitor could greatly benefit patients affected by r/r B-ALL displaying mutant p53.

Currently, several clinical trials are evaluating the use of Chk1 inhibitors for the treatment of patients with either solid tumors or hematological malignances (ClinicalTrials.gov Identifier: NCT02203513 and NCT03495323). While these drugs are still in the early phase of their clinical development, the initial use of the Chk1 antagonist prexasertib in ovarian cancer patients has been associated with good tolerability with the only grade 3-4 adverse events related to decreased white blood cell counts and neutropenia (61).

An alternative approach to the use of Chk1 inhibitors is the combination of chemo- and immune-therapy that was recently described in two different phase 2 clinical trials coupling IO with mini-hyper-CVD in patients with relapsed or refractory ALL (62), or in older patients with newly diagnosed ALL (63). In both cases the trials generated excellent clinical outcomes, although patients experienced different grade 3-4 adverse events including veno-occlusive disease.

In summary, our findings suggest that p53 mutational status may represent a predictive biomarker for IO efficacy and that an approach combining IO and Chk1 inhibition could represent a potential therapeutic approach for patients with r/r B-ALL expressing mutant p53 that are unlikely to benefit from IO monotherapy.

## Author Contributions

ET, MM, AF, PL, and NP performed the experiments. ET, MM, CR, MP, SV, and SS analyzed and interpreted the data. ET and MM designed the experiments and wrote the paper. FS, GP, and AR made a critical revision of paper. LM and FD helped supervise the project. PV conceived the original idea and supervised the project.

### Conflict of Interest Statement

The authors declare that the research was conducted in the absence of any commercial or financial relationships that could be construed as a potential conflict of interest.

## Supplementary Material

Supplemental Figure 1 Chk1 silencing and treatment with Inotuzumab Ozogamicin increases the apoptotic rate of Namalwa cells**. (A)** Namalwa cells transduced with shChk1 or shNS RNAs, were treated for 24 and 48 h with doxycycline to induce the expression of the shRNAs. Protein extracts were blotted for Chk1 to verify the efficacy of the silencing procedure. Actin was used as loading control. **(B)** The indicated cells were induced with doxycycline for 48 hours. After the first 24 h cells were exposed to IO for further 24 h and apoptosis was then evaluated after Annexin V-FITC/7AAD double staining. The indicated percentages show the distribution of necrotic, early and late apoptotic cells after IO treatment. **(C)** Histograms representing the average percentage of Annexin V and 7 AAD-positive cells in the induced condition or after exposure to IO. Columns represent average ± standard deviation of three independent experiments. ^***^*p* < 0.001.

## References

[1] Siegel RL Miller KD Jemal A Cancer statistics, 2015. CA Cancer J Clin. (2015) 65:5–29. 10.3322/caac.21254 25559415

[2] Jaffe ES Harris NL Stein H Isaacson PG. Classification of lymphoid neoplasms: the microscope as a tool for disease discovery. Blood (2008) 112:4384–99. 10.1182/blood-2008-07-077982 19029456 PMC2954680

[3] Rowe JM Buck G Burnett AK Chopra R Wiernik PH Richards SM. Induction therapy for adults with acute lymphoblastic leukemia: results of more than 1500 patients from the international ALL trial: MRC UKALL XII/ECOG E2993. Blood (2005) 106:3760–7. 10.1182/blood-2005-04-1623 16105981

[4] Bassan R Hoelzer D. Modern therapy of acute lymphoblastic leukemia. J Clin Oncol. (2011) 29:532–43. 10.1200/JCO.2010.30.1382 21220592

[5] Terwilliger T Abdul-Hay M. Acute lymphoblastic leukemia: a comprehensive review and 2017 update. Blood Cancer J. (2017) 7:e577. 10.1038/bcj.2017.53 28665419 PMC5520400

[6] Fielding AK. Current treatment of Philadelphia chromosome-positive acute lymphoblastic leukemia. Haematologica (2010) 95:8–12. 10.3324/haematol.2009.015974 20065078 PMC2805747

[7] Farhadfar N Litzow MR. New monoclonal antibodies for the treatment of acute lymphoblastic leukemia. Leuk Res. (2016) 49:13–21. 10.1016/j.leukres.2016.07.009 27521873

[8] Hoelzer D. CD22 monoclonal antibody therapies in relapsed/refractory acute lymphoblastic leukemia. Cancer (2013) 119:2671–4. 10.1002/cncr.28135 23633448

[9] Carnahan J Wang P Kendall R Chen C Hu S Boone T. Epratuzumab, a humanized monoclonal antibody targeting CD22: characterization of *in vitro* properties. Clin Cancer Res. (2003) 9:3982S−3990S. 14506197

[10] McCombs JR Owen SC. Antibody drug conjugates: design and selection of linker, payload and conjugation chemistry. AAPS J. (2015) 17:339–51. 10.1208/s12248-014-9710-8 25604608 PMC4365093

[11] DiJoseph JF Armellino DC Boghaert ER Khandke K Dougher MM Sridharan L. Antibody-targeted chemotherapy with CMC-544: a CD22-targeted immunoconjugate of calicheamicin for the treatment of B-lymphoid malignancies. Blood (2004) 103:1807–14. 10.1182/blood-2003-07-2466 14615373

[12] DiJoseph JF Popplewell A Tickle S Ladyman H Lawson A Kunz A Antibody-targeted chemotherapy of B-cell lymphoma using calicheamicin conjugated to murine or humanized antibody against CD22. Cancer Immunol Immunother. (2005) 54:11–24. 10.1007/s00262-004-0572-2 15693135 PMC11033002

[13] Shor B Gerber HP Sapra P. Preclinical and clinical development of inotuzumab-ozogamicin in hematological malignancies. Mol Immunol. (2015) 67:107–16. 10.1016/j.molimm.2014.09.014 25304309

[14] Zein N Sinha AM McGahren WJ Ellestad GA. Calicheamicin gamma 1I: an antitumor antibiotic that cleaves double-stranded DNA site specifically. Science (1988) 240:1198–201. 3240341 10.1126/science.3240341

[15] Amico D Barbui AM Erba E Rambaldi A Introna M Golay J. Differential response of human acute myeloid leukemia cells to gemtuzumab ozogamicin *in vitro*: role of Chk1 and Chk2 phosphorylation and caspase 3. Blood (2003) 101:4589–97. 10.1182/blood-2002-07-2311 12576328

[16] Takeshita A Shinjo K Yamakage N Ono T Hirano I Matsui H. CMC-544, (inotuzumab ozogamicin) shows less effect on multidrug resistant cells: analyses in cell lines and cells from patients with B-cell chronic lymphocytic leukaemia and lymphoma. Br J Haematol. (2009) 146:34–43. 10.1111/j.1365-2141.2009.07701.x 19388933

[17] Kastan MB Bartek J. Cell-cycle checkpoints and cancer. Nature (2004) 432:316–23. 10.1038/nature03097 15549093

[18] Bieging KT Mello SS Attardi LD. Unravelling mechanisms of p53-mediated tumour suppression. Nat Rev Cancer (2014) 14:359–70. 10.1038/nrc3711 24739573 PMC4049238

[19] Manzella L Stella S Pennisi MS Tirro E Massimino M Romano C. New Insights in thyroid cancer and p53 Family Proteins. Int J Mol Sci. (2017) 18:1325. 10.3390/ijms18061325 28635633 PMC5486146

[20] Miller M Shirole N Tian R Pal D Sordella R. The evolution of TP53 mutations: from loss-of-function to separation-of-function mutants. J Cancer Biol Res. (2016) 4:1091. 28191499 PMC5298884

[21] Xu-Monette ZY Medeiros LJ Li Y Orlowski RZ Andreeff M Bueso-Ramos CE. Dysfunction of the TP53 tumor suppressor gene in lymphoid malignancies. Blood (2012) 119:3668–83. 10.1182/blood-2011-11-366062 22275381 PMC3335376

[22] Salmoiraghi S Montalvo ML Ubiali G Tosi M Peruta B Zanghi P Mutations of TP53 gene in adult acute lymphoblastic leukemia at diagnosis do not affect the achievement of hematologic response but correlate with early relapse and very poor survival. Haematologica (2016) 101:e245–248. 10.3324/haematol.2015.137059 26992948 PMC5013945

[23] Chiaretti S Brugnoletti F Tavolaro S Bonina S Paoloni F Marinelli M. TP53 mutations are frequent in adult acute lymphoblastic leukemia cases negative for recurrent fusion genes and correlate with poor response to induction therapy. Haematologica (2013) 98:e59–61. 10.3324/haematol.2012.076786 23403321 PMC3640132

[24] Kantarjian HM DeAngelo DJ Stelljes M Martinelli G Liedtke M Stock W. Inotuzumab ozogamicin versus standard therapy for acute lymphoblastic leukemia. N Engl J Med. (2016) 375:740–53. 10.1056/NEJMoa1509277 27292104 PMC5594743

[25] Lamb YN. Inotuzumab ozogamicin: first global approval. Drugs (2017) 77:1603–10. 10.1007/s40265-017-0802-5 28819740

[26] Morley NJ Marks DI. Inotuzumab ozogamicin in the management of acute lymphoblastic leukaemia. Expert Rev Anticancer Ther. (2016) 16:159–64. 10.1586/14737140.2016.1131614 26654587

[27] Giallongo C Parrinello N Tibullo D La Cava P Romano A Chiarenza A. Myeloid derived suppressor cells, (MDSCs) are increased and exert immunosuppressive activity together with polymorphonuclear leukocytes, (PMNs) in chronic myeloid leukemia patients. PLoS ONE (2014) 9:e101848. 10.1371/journal.pone.0101848 25014230 PMC4094386

[28] Buffa P Romano C Pandini A Massimino M Tirro E Di Raimondo F. BCR-ABL residues interacting with ponatinib are critical to preserve the tumorigenic potential of the oncoprotein. FASEB J. (2014) 28:1221–36. 10.1096/fj.13-236992 24297701

[29] Massimino M Vigneri P Fallica M Fidilio A Aloisi A Frasca F. IRF5 promotes the proliferation of human thyroid cancer cells. Mol Cancer (2012) 11:21. 10.1186/1476-4598-11-21 22507190 PMC3444366

[30] Massimino M Consoli ML Mesuraca M Stagno F Tirro E Stella S. IRF5 is a target of BCR-ABL kinase activity and reduces CML cell proliferation. Carcinogenesis (2014) 35:1132–43. 10.1093/carcin/bgu013 24445143

[31] Dijoseph JF Dougher MM Armellino DC Evans DY Damle NK. Therapeutic potential of CD22-specific antibody-targeted chemotherapy using inotuzumab ozogamicin, (CMC-544) for the treatment of acute lymphoblastic leukemia. Leukemia (2007) 21:2240–5. 10.1038/sj.leu.2404866 17657218

[32] Stella S Tirro E Conte E Stagno F Di Raimondo F Manzella L. Suppression of survivin induced by a BCR-ABL/JAK2/STAT3 pathway sensitizes imatinib-resistant CML cells to different cytotoxic drugs. Mol Cancer Ther. (2013) 12:1085–98. 10.1158/1535-7163.MCT-12-0550 23536723

[33] Farrell PJ Allan GJ Shanahan F Vousden KH Crook T. p53 is frequently mutated in Burkitt's lymphoma cell lines. EMBO J. (1991) 10:2879–87. 1915267 10.1002/j.1460-2075.1991.tb07837.x PMC452998

[34] Branzei D Foiani M. Regulation of DNA repair throughout the cell cycle. Nat Rev Mol Cell Biol. (2008) 9:297–308. 10.1038/nrm2351 18285803

[35] Preyer M Vigneri P Wang JY. Interplay between kinase domain autophosphorylation and F-actin binding domain in regulating imatinib sensitivity and nuclear import of BCR-ABL. PLoS ONE (2011) 6:e17020. 10.1371/journal.pone.0017020 21347248 PMC3037956

[36] Wang Y Ji P Liu J Broaddus RR Xue F Zhang W. Centrosome-associated regulators of the G(2)/M checkpoint as targets for cancer therapy. Mol Cancer (2009) 8:8. 10.1186/1476-4598-8-8 19216791 PMC2657106

[37] Chen Y Poon RY. The multiple checkpoint functions of CHK1 and CHK2 in maintenance of genome stability. Front Biosci. (2008) 13:5016–29. 18508566 10.2741/3060

[38] Ou YH Chung PH Sun TP Shieh SY. p53 C-terminal phosphorylation by CHK1 and CHK2 participates in the regulation of DNA-damage-induced C-terminal acetylation. Mol Biol Cell (2005) 16:1684–95. 10.1091/mbc.E04-08-0689 15659650 PMC1073652

[39] Hirose Y Berger MS Pieper RO. Abrogation of the Chk1-mediated G(2) checkpoint pathway potentiates temozolomide-induced toxicity in a p53-independent manner in human glioblastoma cells. Cancer Res. (2001) 61:5843–9. 11479224

[40] Husain A Yan XJ Rosales N Aghajanian C Schwartz GK Spriggs DR. UCN-01 in ovary cancer cells: effective as a single agent and in combination with cis-diamminedichloroplatinum(II)independent of p53 status. Clin Cancer Res. (1997) 3:2089–97. 9815601

[41] Levesque AA Fanous AA Poh A Eastman A. Defective p53 signaling in p53 wild-type tumors attenuates p21waf1 induction and cyclin B repression rendering them sensitive to Chk1 inhibitors that abrogate DNA damage-induced S and G2 arrest. Mol Cancer Ther. (2008) 7:252–62. 10.1158/1535-7163.MCT-07-2066 18281511

[42] Donzelli M Draetta GF. Regulating mammalian checkpoints through Cdc25 inactivation. EMBO Rep. (2003) 4:671–7. 10.1038/sj.embor.embor887 12835754 PMC1326326

[43] Duthu A Debuire B Romano J Ehrhart JC Fiscella M May E. p53 mutations in Raji cells: characterization and localization relative to other Burkitt's lymphomas. Oncogene (1992) 7:2161–7. 1437144

[44] Xu J Reumers J Couceiro JR De Smet F Gallardo R Rudyak S. Gain of function of mutant p53 by coaggregation with multiple tumor suppressors. Nat Chem Biol. (2011) 7:285–95. 10.1038/nchembio.546 21445056

[45] Walton MI Wilson SC Hardcastle IR Mirza AR Workman P. An evaluation of the ability of pifithrin-alpha and -beta to inhibit p53 function in two wild-type p53 human tumor cell lines. Mol Cancer Ther. (2005) 4:1369–77. 10.1158/1535-7163.MCT-04-0341 16170029

[46] Perdrix A Najem A Saussez S Awada A Journe F Ghanem G PRIMA-1 and PRIMA-1(Met), (APR-246): From Mutant/Wild Type p53 Reactivation to unexpected mechanisms underlying their potent anti-tumor effect in combinatorial therapies. Cancers (2017) 9:E172 10.3390/cancers9120172 29258181 PMC5742820

[47] Stengel A Schnittger S Weissmann S Kuznia S Kern W Kohlmann A. TP53 mutations occur in 15.7% of ALL and are associated with MYC-rearrangement, low hypodiploidy, and a poor prognosis. Blood (2014) 124:251–8. 10.1182/blood-2014-02-558833 24829203

[48] Xu P Liu X Ouyang J Chen B. TP53 mutation predicts the poor prognosis of non-Hodgkin lymphomas: Evidence from a meta-analysis. PLoS ONE (2017) 12:e0174809. 10.1371/journal.pone.0174809 28369138 PMC5378372

[49] Iacobucci I Di Rora AG Falzacappa MV Agostinelli C Derenzini E Ferrari A. *In vitro* and *in vivo* single-agent efficacy of checkpoint kinase inhibition in acute lymphoblastic leukemia. J Hematol Oncol. (2015) 8:125. 10.1186/s13045-015-0206-5 26542114 PMC4635624

[50] Garcia-Alonso S Ocana A Pandiella A. Resistance to antibody-drug conjugates. Cancer Res. (2018) 78:2159–65. 10.1158/0008-5472.CAN-17-3671 29653942

[51] de Vries JF Zwaan CM De Bie M Voerman JS den Boer ML van Dongen JJ. The novel calicheamicin-conjugated CD22 antibody inotuzumab ozogamicin, (CMC-544) effectively kills primary pediatric acute lymphoblastic leukemia cells. Leukemia (2012) 26:255–64. 10.1038/leu.2011.206 21869836

[52] Bartek J Lukas J. Chk1 and Chk2 kinases in checkpoint control and cancer. Cancer Cell (2003) 3:421–9. 12781359 10.1016/s1535-6108(03)00110-7

[53] Agarwal ML Agarwal A Taylor WR Stark GR. p53 controls both the G2/M and the G1 cell cycle checkpoints and mediates reversible growth arrest in human fibroblasts. Proc Natl Acad Sci USA. (1995) 92:8493–7. 7667317 10.1073/pnas.92.18.8493 PMC41183

[54] Carrassa L Damia G. DNA damage response inhibitors: Mechanisms and potential applications in cancer therapy. Cancer Treat Rev. (2017) 60:139–51. 10.1016/j.ctrv.2017.08.013 28961555

[55] Giallongo C Tibullo D La Cava P Branca A Parrinello N Spina P. BRIT1/MCPH1 expression in chronic myeloid leukemia and its regulation of the G2/M checkpoint. Acta Haematol. (2011) 126:205–10. 10.1159/000329911 21934293

[56] Krenning L Feringa FM Shaltiel IA van den Berg J Medema RH. Transient activation of p53 in G2 phase is sufficient to induce senescence. Mol Cell (2014) 55:59–72. 10.1016/j.molcel.2014.05.007 24910099

[57] George B Kantarjian H Jabbour E Jain N. Role of inotuzumab ozogamicin in the treatment of relapsed/refractory acute lymphoblastic leukemia. Immunotherapy (2016) 8:135–43. 10.2217/imt.15.108 26780449 PMC5618942

[58] Prokop A Wrasidlo W Lode H Herold R Lang F Henze G. Induction of apoptosis by enediyne antibiotic calicheamicin thetaII proceeds through a caspase-mediated mitochondrial amplification loop in an entirely Bax-dependent manner. Oncogene (2003) 22:9107–20. 10.1038/sj.onc.1207196 14647446

[59] Inaba H Greaves M Mullighan CG. Acute lymphoblastic leukaemia. Lancet (2013) 381:1943–55. 10.1016/S0140-6736(12)62187-4 23523389 PMC3816716

[60] Schuler F Weiss JG Lindner SE Lohmuller M Herzog S Spiegl SF. Checkpoint kinase 1 is essential for normal B cell development and lymphomagenesis. Nat Commun. (2017) 8:1697. 10.1038/s41467-017-01850-4 29167438 PMC5700047

[61] Lee JM Nair J Zimmer A Lipkowitz S Annunziata CM Merino MJ. Prexasertib, a cell cycle checkpoint kinase 1 and 2 inhibitor, in BRCA wild-type recurrent high-grade serous ovarian cancer: a first-in-class proof-of-concept phase 2 study. Lancet Oncol. (2018) 19:207–15. 10.1016/S1470-2045(18)30009-3 29361470 PMC7366122

[62] Jabbour E Ravandi F Kebriaei P Huang X Short NJ Thomas D Salvage chemoimmunotherapy with inotuzumab ozogamicin combined with mini-Hyper-CVD for Patients with relapsed or refractory philadelphia chromosome-negative acute lymphoblastic leukemia: a phase 2 clinical trial. JAMA Oncol. (2017) 4:230–4. 10.1001/jamaoncol.2017.2380 PMC5838597 28859185

[63] Kantarjian H Ravandi F Short NJ Huang X Jain N Sasaki K. Inotuzumab ozogamicin in combination with low-intensity chemotherapy for older patients with Philadelphia chromosome-negative acute lymphoblastic leukaemia: a single-arm, phase 2 study. Lancet Oncol. (2018) 19:240–8. 10.1016/S1470-2045(18)30011-1 29352703 PMC11537312

