# Supplementary figures and images for: Chk1 Inhibition Restores Inotuzumab Ozogamicin Citotoxicity in CD22-Positive Cells Expressing Mutant p53
Source: Front Oncol. 2019 Feb 18;9:57. doi: 10.3389/fonc.2019.00057 (PMC6387953; doi:10.3389/fonc.2019.00057)

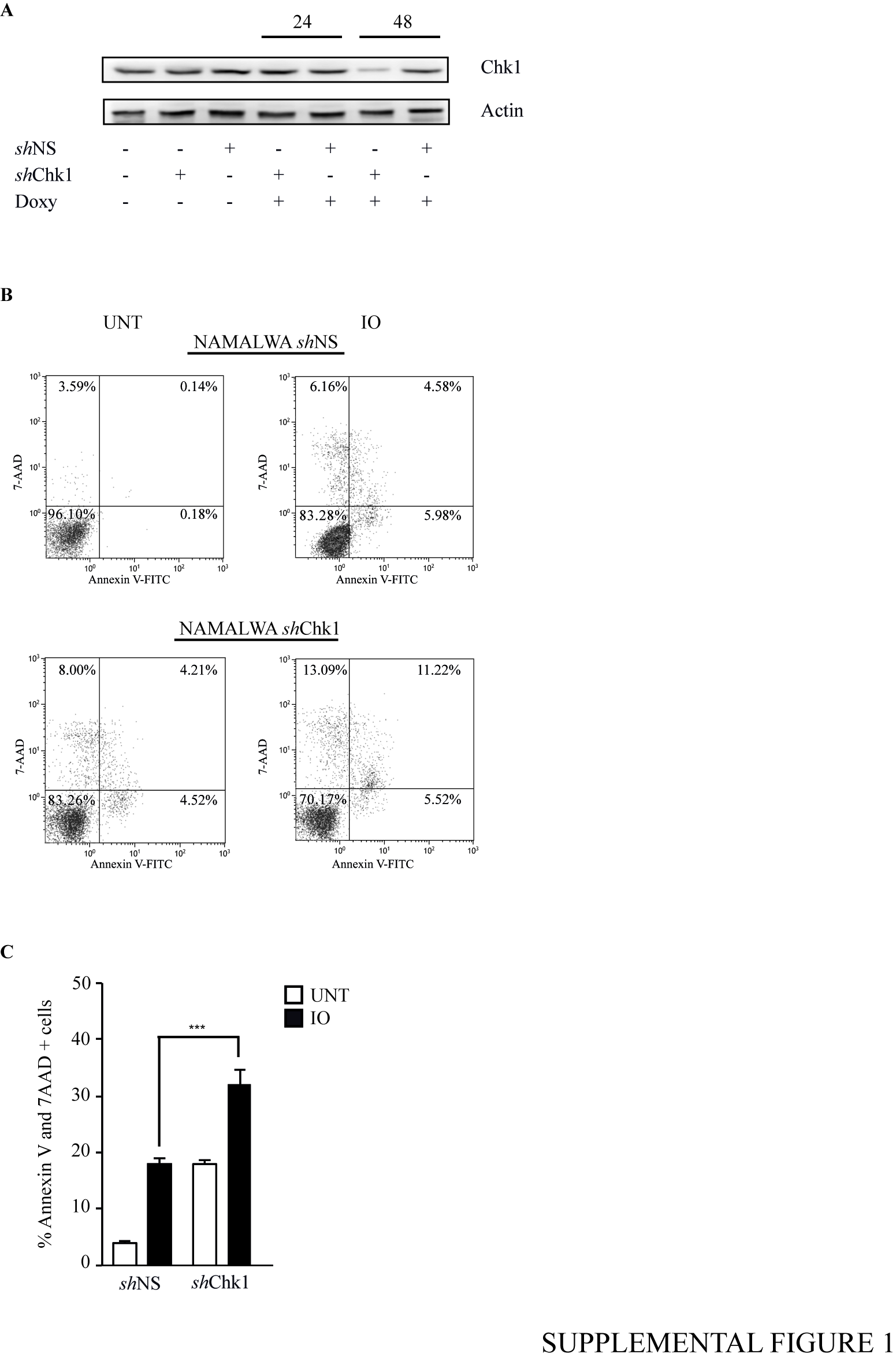

Supplement: Supplemental Figure 1 — Chk1 silencing and treatment with Inotuzumab Ozogamicin increases the apoptotic rate of Namalwa cells. (A) Namalwa cells transduced with shChk1 or shNS RNAs, were treated for 24 and 48 h with doxycycline to induce the expression of the shRNAs. Protein extracts were blotted for Chk1 to verify the efficacy of the silencing procedure. Actin was used as loading control. (B) The indicated cells were induced with doxycycline for 48 hours. After the first 24 h cells were exposed to IO for further 24 h and apoptosis was then evaluated after Annexin V-FITC/7AAD double staining. The indicated percentages show the distribution of necrotic, early and late apoptotic cells after IO treatment. (C) Histograms representing the average percentage of Annexin V and 7 AAD-positive cells in the induced condition or after exposure to IO. Columns represent average ± standard deviation of three independent experiments. ***p < 0.001. [file Image_1.TIF]
